# The potential of soil microbial communities to transform deoxynivalenol in agricultural soils—a soil microcosm study

**DOI:** 10.1007/s12550-024-00526-5

**Published:** 2024-03-20

**Authors:** Kilian G. J. Kenngott, Katherine Muñoz

**Affiliations:** Institute for Environmental Sciences (iES) Landau, RPTU Kaiserslautern-Landau, Fortstraße 7, Landau, 76829 Rhineland-Palatinate Germany

**Keywords:** DON, 3-epi-DON, 3-keto-DON, Soil microbial transformation, Soil microcosm

## Abstract

Infestation of cereal fields with toxigenic *Fusarium* species is identified as an environmental source for the mycotoxin deoxynivalenol (DON). During rain events, DON may be washed off from infested plants and enter the soil, where microbial transformation may occur. Although some studies showed DON transformation potential of soil microbial communities in liquid soil extracts, these findings can not be transferred to environmental conditions. Accordingly, microbial transformation of DON in soil has to be investigated under realistic conditions, e.g., microcosms mimicking field situations. In this study, we investigated the potential of soil microbial communities to transform DON in six different agricultural soils at two levels (0.5 and 5 µg g^–1^). The dissipation and the formation of transformation products were investigated in a period of 35 days and compared to a sterilized control. In addition, we measured soil respiration and applied the phospholipid-derived fatty acid (PLFA) analysis to assess whether soil microbial community characteristics are related to the microbial transformation potential. Dissipation of DON in non-sterilized soils was fast (50% dissipation within 0.6–3.7 days) compared to the sterile control where almost no dissipation was observed. Thus, dissipation was mainly attributed to microbial transformation. We verified that small amounts of DON are transformed to 3-keto-deoxynivalenol (3-keto-DON) and 3-epi-deoxynivalenol (3-epi-DON), which were not detectable after 16-day incubation, indicating further transformation processes. There was a trend towards faster transformation in soils with active and large microbial communities and low fungi-to-bacteria ratio.

**Supplementary Information:**

The online version contains supplementary material available at 10.1007/s12550-024-00526-5.

## Introduction

The mycotoxin deoxynivalenol (DON) is often found in cereal crops (European Food Safety Authority 2013) as a consequence of the infestation with pathogenic *Fusarium* species. Due to the good water solubility and low affinity to natural organic matter (Schenzel et al. 2012b), DON can be easily mobilized from infested plants (Schenzel et al. 2012a) or harvest residues during rain events, being found in soil and soil leachate (Meyer-Wolfarth et al. 2021), in drainage water of fields (Schenzel et al. 2012a), and in surface waters (Schenzel et al. 2012c; Kolpin et al. 2014). However, mobilized DON will initially enter the soil, and not all rain events are heavy enough for further mobilization processes. This provides an opportunity for transformation processes of DON in soil with the potential to mitigate environmental DON levels.

Although DON is chemically stable in a wide range of physical and chemical conditions (Joint FAO/WHO Expert Committee on Food Additives 2001; Mishra et al. 2014), the dissipation of DON in soil has been reported in some studies and is therefore associated with biological transformation processes. Meyer et al. (2021) showed decreasing DON levels from up to 21 ng g^–1^ to background levels within 11 weeks in a field study. In other experiments, DON was incorporated to soil microcosms as part of harvest residues (10 µg g^–1^ in straw, 10.5 µg g^–1^ in maize stubbles) and levels dropped by 96 and 79% within 2 and 6 weeks, respectively (Abid et al. 2021; Meyer-Wolfarth et al. 2021). However, these experiments included various edaphic groups, i.e., earthworms, nematodes, springtails, and soil microbial communities, and the respective contribution to disappearance is not yet fully understood.

Studies assessing the potential of various soil microbial communities indicate that the respective DON transformation potential may be rare and only individual soils are able to degrade DON. In a study by Völkl et al. (2004), 40 mixed soil cultures and 287 pure cultures from arable soils were assessed, but none of the microbial cultures metabolized DON under the applied experimental conditions. Similarly, only one out of five composite samples (165 agricultural soils) was observed to transform DON in a study by Islam et al. (2012). One reason may be that single genera or species of the soil microbial community are responsible for DON transformation but can not survive the isolation process and laboratory conditions in aqueous mineral media (Ward et al. 1990; Völkl et al. 2004). Therefore, the results obtained under these laboratory conditions can not be transferred to natural soil conditions, and in situ microbial transformation in soil remains unexplored.

Other studies confirmed the DON transformation potential of some soil microbial communities and identified different transformation paths in solution. Islam et al. (2012) showed 100% de-epoxidation of 50 µg mL^–1^ DON by a mixed soil microbial community culture after 6 days in mineral salt + bacto peptone medium. In a follow-up study, the same culture de-epoxidized several trichothecenes under aerobic and anaerobic conditions and even high levels of 500 µg mL^–1^ DON (Ahad et al. 2017). Another transformation path, i.e., epimerization of DON, was shown for the soil bacteria *Nocardioides* WSN05-2 (Ikunaga et al. 2011), *Devosia insulae* A16 (Wang et al. 2019), and a mixed culture of soil bacteria (Zhai et al. 2019), which were able to transform various concentrations of DON (20–1000 µg mL^–1^) to 3-epi-DON within 2–10 days when DON was the only carbon source. The soil bacterium *Deviosa mutans* 17-2-E-8 was able to epimerize up to 89% of 100 µg mL^–1^ DON within 3 days even when DON was not the only carbon source (He et al. 2016). However, these studies mainly focus on isolating bacterial strains from soil with the aim to use them in cereal food processing, and no conclusions regarding transformation processes in soil can be drawn.

Contrary to studies, where DON transformation was investigated using the cultivable part of the soil microbial community, we focused on the transformation of available DON by the whole soil microbiome in soil microcosms. In the first line, we aim to assess the potential of soils for mitigating DON occurrence in the environment measured as the dissipation of dissolved DON in soil. Furthermore, we assess the contribution of biotic (microbial) and abiotic transformation to the observed dissipation of DON. We hypothesize that soil microbial communities are able to degrade DON in soil and that the transformation depends on general soil microbial community characteristics, i.e., activity and biomass as it has been shown in studies assessing herbicide dissipation (Anderson 1984). Since soil bacteria are more efficient users of mobile dissolved carbon sources (Marschner and Kalbitz 2003; Kramer and Gleixner 2008), we hypothesize that bacteria are the main contributors to DON transformation, and therefore, not only the size (biomass) of the community, but also the soil microbial community structure influences the transformation. Finally, we expect that the transformation of DON will result in the formation of 3-keto-DON and 3-epi-DON as these compounds were found in studies using microbial communities and species isolated from soil (Yao and Long 2020). To test our hypotheses, we incubated six soils spiked with DON (0.5 and 5 µg g^–1^) for up to 35 days and measured the decline in DON levels. Sterile soil samples were included to asses abiotic transformation. We measured glucose-induced respiration and applied the phospholipid-derived fatty acid (PLFA) analysis to assess the soil microbial activity and structure (biomass: total PLFA; Structure: total bacteria, total fungi, fungi-to-bacteria ratio), respectively.

## Materials and methods

### Chemicals

Methanol and formic acid (LC-MS grade) for mobile phase, soil extraction, and standard preparation were supplied by Fisher Scientific (Schwerte, Germany). Ultrapure water was used throughout the whole study (Milli-Q-purification system, 18.2 M$\Omega$ c$^{-1}$m, EASYpure II, Millipore, Bedford, MA, USA). Stock solution of DON was prepared by dilution of 5 mg DON (Sigma-Aldrich, St. Louis, USA) in 20 mL of water. DON stock solution was used for the preparation of spiking solutions at 25 and 250 µg mL^–1^ and for calibration. Isotopically labeled standard ^13^C_15_-deoxynivalenol dissolved in acetonitrile was supplied by Biopure^TM^, Romer Labs (Butzbach, Germany), and diluted to 250 ng mL^–1^ in methanol. 3-keto-DON standard for calibration was supplied by Triplebond (Guelph, Canada). No reference standard was available for 3-epi-DON, but one peak was putatively assigned to 3-epi-DON based on the shorter retention time which has been reported in other studies (Vanhoutte et al. 2017; Zhai et al. 2019) and based on the mass signals fitting with the signals of DON.

Glucose for soil respiration measurement as well as methanol, chloroform, and acetone for PLFA extraction were purchased by Carl Roth (Karlsruhe, Germany). A 0.25 M methanolic trimethylsulfonium hydroxide solution for PLFA transesterification was purchased by Sigma-Aldrich (St. Louis, USA). The quantitative 37-component fatty acid methyl ester mix (Supelco, Bellefonte, PA, USA) was spiked with additional components i15:0, i17:0, 16:1$\omega$5c, and 18:1$\omega$7c (Larodan AB, Solna, Sweden) and used for quantification. A 250 µg mL^-1^ solution of 1,2-dimyristoyl-sn-glycero-3-phosphocholine (Sigma-Aldrich, St. Louis, USA) in methanol was used as an internal standard for PLFA analysis.

### Soil selection and pre-incubation

Soils were selected in order to cover a range of soil physicochemical properties, namely texture, pH, and total carbon content (TC) (Table 1) and land management. Three soils (JA-cornfield, F03P02, and F07P01) were sampled from maize fields. F03P02 and F07P01 were exposed to DON in the past as shown in a previous study (Kenngott et al. 2022). Three soils (Refesol03G, Refesol04A, Refesol05G) were supplied by Fraunhofer IME (Schmallenberg, Germany; order codes 03-G, 04-A, 05-G), further referred to as reference soils. The reference soils are recognized by the German Federal Environmental Agency for application in biological tests and for determination of decomposition and sorption processes. The soils Refesol03G and Refesol05G originate from grassland, and Refesol04A was sampled from arable land. No cereal plants have been grown on the reference soils since September 2019.

F03P02 and F07P01 were sampled in September 2019 from corn field topsoil (0–5 cm soil depth) as described in Kenngott et al. (2022) and stored at $-$20 ${}^{\circ }\text {C}$ until analysis. Prior to analysis, soils were unfrozen, sieved (2 mm), homogenized, and stored in darkness at 21 ${}^{\circ }\text {C}$ for 4 and 1 week for pre-incubation, respectively. JA-cornfield soil was sampled in August 2021 from a corn field topsoil (0–20 cm soil depth), homogenized, and stored outdoor in a plastic barrel with air exchange. A fresh subsample was taken and sieved (2 mm) the day before soil sample pre-incubation. Reference soils were sampled 1 week prior to soil sample pre-incubation.

The targeted water holding capacity (WHC) during the experiment was 50%. Therefore, all soils were initially adjusted to 40% WHC for pre-incubation and the remaining 10% were added in the subsequent DON spiking step. Moistened soils were homogenized and aliquots equivalent to 2.5 g soil dry weight were weighed in 15 mL plastic centrifuge tubes. Screw caps were loosely placed on each tube to reduce evaporation and protect the soil from dust or contamination but allowing air exchange. All tubes were pre-incubated in darkness at 21 ${}^{\circ }\text {C}$ for 4 days to allow microbial communities to adapt to the experimental conditions. Sterile references were produced by autoclaving tubes with soil aliquots for 1 h at 121 ${}^{\circ }\text {C}$. Autoclaving was repeated 3 days later to eliminate colonies from intact spores. Aliquots of sterilized soils (1 g) were placed on agar medium plates to verify the absence of colony-forming units and confirm sterile conditions after autoclaving. Screw caps of sterilized samples were tightly closed after autoclaving to avoid microbial contamination.

**Table 1:** Soil physicochemical parameters: texture fractions, water holding capacity (WHC) in gram water per gram soil dry weight, pH measured in 0.01 M CaCl_2_ soil extract (1:5, v:v), total carbon content (TC), cultivation

Soil	Sand %	Silt %	Clay %	WHC g g^–1^	pH	TC %	Cultivation
F03P02	31.0	65.0	4.0	0.24	7.10	0.89	Maize field
F07P01	22.5	22.5	55.0	0.39	7.50	2.40	Maize field
JA-cornfield	21.3	46.0	32.7	0.50	7.04	1.87	Maize field
Refesol03G	17.7	57.5	24.8	0.73	5.97	3.01	Grassland
Refesol04A	79.7	14.9	5.4	0.35	5.11	3.04	Agricultural
Refesol05G	21.9	59.0	19.1	0.67	5.37	1.92	Grassland

### DON dissipation experiment

After the pre-incubation, soils in tubes were spiked with 50 $\upmu$L of DON spiking solutions (25 and 250 µg mL^–1^) to reach levels of 0.5 and 5 µg g^–1^ soil dry weight. Tubes spiked with pure water served as control. Additional water was added to adjust the WHC to 50%. Screw caps were again loosely placed on each tube to reduce evaporation and protect the soil from dust or contamination but allowing air exchange. The tubes were then further incubated in darkness at 21 ${}^{\circ }\text {C}$ until sampling.

Sterile soils in tubes were spiked with 50 $\upmu$L of filtered (0.2 $\upmu$m) high level DON spiking solution (250 µg mL^–1^), and WHC was adjusted to 50% using autoclaved water. Screw caps of sterilized tubes were tightly closed during incubation. To ensure air exchange, the caps were perforated with a sharp cannula, and the hole was immediately covered with medical tape to avoid microbial contamination.

After 0, 1, 2, 4, 7, 11, 16, and 35 days of incubation, three tubes of each soil at each spiking level (3 replicates × 6 soils × 2 levels × 8 sampling times = 288 samples) were sampled from the incubator and frozen at $-$20 ${}^{\circ }\text {C}$ until further processing. At the same time, one control sample was taken and stored likewise (1 replicate × 6 soils × 1 level × 8 sampling times = 48 samples). Since no degradation was expected in sterile soil, three sterilized tubes of each soil were taken less frequently after 1, 7, 16, and 35 days (3 replicate × 6 soils × 1 level × 4 sampling times = 72 sterile samples) and stored at $-$20 ${}^{\circ }\text {C}$.

### Extraction and measurement of DON and DON transformation products

After freeze drying to constant weight at $-$40 ${}^{\circ }\text {C}$, the samples were mixed with 5 mL of 10% methanolic water solution for 30 min at 180 rpm in an orbital shaker. After 15 min in an ultrasonic bath, samples were centrifuged for 15 min at 2190 g. Supernatands were filtered through 0.2 $\upmu$m PET syringe filters, and aliquots of 90 $\upmu$L were mixed with 10 $\upmu$L of internal standard solution.

Measurement system was a liquid chromatography high-resolution mass spectrometry Thermo Scientific^®^ equipped with a quaternary Accela^®^ pump, an Exactive^®^ Orbitrap MS detector (Thermo Fisher Scientific, Waltham, USA) and a Hypersil GOLD™ C18 column with dimensions 100 × 2.1 mm and 1.9 $\upmu$m particle size (Thermo Fisher Scientific, Waltham, USA). Mobile phase consisted of methanol (Solvent A) and water (Solvent B) each conditioned with 0.1% formic acid. Gradient program with constant flow rate (0.2 mL min$^{-1}$) was as follows: 0–2 min 19% Solvent A; 2–6 min 19–100% Solvent A; 6–11 min 100% Solvent A; 11–12 min 100–19% Solvent A; 12–15 min 19% Solvent A. Injection volume was 10 $\upmu$L. Electron spray ionization was performed in negative mode at a capillary temperature of 275 ${}^{\circ }\text {C}$. Electronic settings were as follows: spray voltage 4 kV, capillary voltage 25 V, tube lens voltage 75 V, and skimmer voltage 14 V.

The target masses of DON, 3-keto-DON, and ^13^C_15_-DON were selected as the masses with the highest signal intensity of pure compound in mobile phase. A suspicious peak with the same mass signal but a slightly shorter retention time compared to DON was putatively assigned to 3-epi-DON. The same target mass and calibration as for DON were used for 3-epi-DON detection and quantification. Target masses with respective adduct and retention time used for high-resolution mass spectrometry detection are shown in Table 2, and example chromatograms are presented in the Appendix (Figs. A1 and A2). All targets were measured in negative mode as the formic acid adduct.

**Table 2:** Overview of retention time, molecular mass, and exact masses of the most intensive ions used for quantification

Analyte	Retention time	Molecular weight	Adduct	Target Mass
	(min)	(Da)		(*m/z*)
3-epi-DON	3	296.16	[M+COOH]^–^	341.1242
DON	3.8	296.16	[M+COOH]^–^	341.1242
3-keto-DON	6.3	294.30	[M+COOH]^–^	339.1079
^13^C_15_-DON	3.8	311.21	[M+COOH]^–^	356.1750

### Soil microbial parameters

Glucose-induced respiration was measured using the CarbOBot system (CarbOBot, prw Electronics, Berlin, Germany). The measurement system is described in detail elsewhere (Heitkötter 2018). Briefly, soil samples amended with glucose are placed in closed containers where the released CO_2_ is allowed to react with KOH solution. The change in electric conductivity of the KOH solution is used to calculate the released CO_2_ carbon (CO_2_-C). Empty containers were used as a temperature reference.

Subsamples of 10 g of pre-incubated soils (40% WHC, 8 days in darkness at 21 ${}^{\circ }\text {C}$) were weighed in Petri dishes, and glucose solution (150 mg mL^–1^) were dripped on the soil to adjust to 50% WHC and achieve 30 mg mL^–1^ soil water as recommended by West and Sparling (1986). Additionally, further subsamples were similarly spiked with pure water (basal respiration). Triplicaes were used for each soil and respiration measurement (basal and glucose induced). Mean respiration, i.e., CO_2_-C release per time, after 6.0 ± 0.2 h was used to assess soil microbial activity.

The PLFA analysis was adapted from Kenngott et al. (2021). Subsamples of 2 g from each soil were weighed in glass culture tubes and pre-incubated as described in Section 2.3. Triplicates of each soil were spiked to 5 µg g^–1^ DON at 50% WHC, incubated together with the transformation experiment samples and taken 4 days after DON application. Samples were frozen and stored at $-$20 ${}^{\circ }\text {C}$ until further processing. Prior to the PLFA analysis, soil samples were freeze-dried to a constant weight at $-$40 ${}^{\circ }\text {C}$. The extraction was performed in accordance with Bligh and Dyer (1959) and White et al. (1979). Briefly, samples were spiked with 50 $\upmu$L of internal standard solution, and lipids were extracted with a mixture of phosphate buffer, chloroform and methanol (1.6:2:4 v/v/v). The samples were shaken for 2 h and centrifuged for 15 min at 716 g. The supernatants were transferred to a fresh glass tube and mixed with 1.6 mL of phosphate buffer and 2 mL of chloroform to allow phase separation. The upper layer was discarded, and the lower layer was dried under a gentle nitrogen stream at 30 ${}^{\circ }\text {C}$. Lipids were redissolved in 1 mL chloroform and transferred to solid-phase extraction cartridges (Chromabond Easy, Marchery-Nagel, Düren, Germany). Phospholipids were separated from the neutral and glyco lipids using 5 mL chloroform and 10 mL acetone and eluted with 6 mL methanol. The eluate was evaporated and redissolved in 0.2 mL methanol. The lipids were transesterified by mixing 20 $\upmu$L of sample extract with a 0.25 M methanolic trimethylsulfonium hydroxide solution (Butte 1983; Gómez-Brandón et al. 2010). The samples were analyzed by gas chromatography with a flame ionization detector (Trace GC Ultra, Thermo Fisher Scientific, Waltham, USA) equipped with a 30-m FAMEWAX column (Restek Corporation, Bellefonte, PA, USA) and a glass wool liner. A sample volume of 1 $\upmu$L was injected at 120 ${}^{\circ }\text {C}$ splitless. Carrier gas was He at 1.4 mL min$^{-1}$ constant column flow. The oven program was 2.5 min at 80 ${}^{\circ }\text {C}$, with 34 ${}^{\circ }\text {C}$ min$^{-1}$ to 170 ${}^{\circ }\text {C}$ and hold for 6 min, with 12 ${}^{\circ }\text {C}$ min$^{-1}$ to 245 ${}^{\circ }\text {C}$ and hold for 3.5 min, with 12 ${}^{\circ }\text {C}$ min$^{-1}$ to 250 ${}^{\circ }\text {C}$ and hold for 2 min. The following PLFAs were used as biomarkers for soil microbial community groups: i15:0, i17:0 (Gram-positive; Ratledge (1997)); 16:1$\omega$7, 18:1$\omega$9c, 18:1$\omega$7c (Gram-negative; Ratledge (1997)); 16:1$\omega$5c (arbuscular mycorrhizal fungi; Olsson et al. (1995); van Aarle and Olsson (2003)); 18:2$\omega$6c (Fungi; Frostegård and Bååth (1996); Kaiser et al. (2010)). The sum of all quantifiable PLFA was used as a proxy for soil microbial biomass.

### Soil physicochemical parameters

Soil physicochemical parameters of reference soils were provided by the supplier. Soil pH was measured electrochemically in a 0.01 molL$^{-1}$ CaCl_2_ solution (1:5 ratio, soil to solution) of field fresh soil. Soil texture was determined according to DIN 19682-2 (F03P02, F07P01, Kenngott et al. (2022)) and ASTM D422–63 (2007) (JA-cornfield). Total carbon content was measured by dry combustion in tin foils using an elemental analyzer (vario MicroCUBE, Elementar Analysensysteme GmbH, Langenselbold, Germany). Total carbon can be assumed as soil organic carbon, because of the low amount of carbonates in agricultural soils. The WHC was defined as the amount of water that is retained by the soil (g g^-1^) against gravity after 1 h of saturation with water.

### Data evaluation

Data evaluation and presentation were performed using the software R (R Core Team 2018) including the package “data.table” and “ggplot2” (Dowle and Srinivasan 2021; Wickham 2016). Model assumptions (i.e., normality and homoscedasticity of data and linear model residuals) were evaluated with QQ and residual vs. fitted plots (Zuur et al. 2010). When linear models for calibration showed heteroscedasticity of residuals, a weighted least squares linear regression was performed. The best weighting factor for weighted least squares linear regression was selected using the function *weight_select*, and the regression was performed using the function *calibration* from the package “envalysis” (Steinmetz 2023). The limit of quantification was calculated as the standard error of ten measured replicates at a near limit of quantification concentration (0.005 µg mL^–1^) multiplied by 10 (Magnusson and Örnemark 2014). To correct for matrix effects, the peak of DON was divided by the peak area of the isotopically labeled internal standard for each measurement. Simple first-order models were fitted on the residual levels using the function *nls* from the package “minpack.lm” (Elzhov et al. 2022). The fitted models were used to estimate the time when 50% of the initially applied level disappeared (DT_50_).

Prior to the statistical evaluation of spiking level and microbial community effects, residual levels were set relative to the spiking level to account for different orders of magnitude in spiking levels, further referred to as relative residual DON levels. Additionally, values were rank transformed to achieve normal distribution. The difference in relative residual DON levels was tested via the Wilcoxon rank sum test.

The effect of predictor variable “microbial respiration” on relative residual DON levels was tested via linear mixed effect models using the function *lmer* from the package “lmerTest” (Kuznetsova et al. 2017). The effect of the predictor variables “soil microbial biomass” and “fungi-to-bacteria ratio” as well as the interaction was tested accordingly in a second model. To account for the variability caused by “spiking level,” this factor was included in the models as a random effect.

Degrees of freedom and *F*-statistics were performed via Kenward-Roger approximation according to Halekoh and Højsgaard (2014) using the package “lmerTest” and “pbkrtest” (Kuznetsova et al. 2017; Halekoh and Højsgaard 2014).

## Results

### Method performance

The linearity of the analytical method was assessed as the adjusted R^2^ of the calibration with ten concentrations in a range of 0.005–5 µg mL^–1^ which was 0.99 for DON. Since the residuals of the calibration showed heteroscedasticity, a weighing factor of 1/y^2^ was included (Almeida et al. 2002). The limit of quantification for DON was 0.006 µg mL^–1^ corresponding to 0.013 µg g^–1^ soil dry weight. The recovery was estimated as the fraction of the initially spiked DON amount which was quantified in the first sampling (day 0, up to 4.5 h after spiking). Overall mean recovery was 91 ± 23%. The high variation was mainly attributed to fast degradation leading to lower recovery and one erroneous spiking causing high recovery, described in the next chapter.

3-keto-DON was calibrated with five concentrations in the range of 0.004–2 µg mL^–1^. The adjusted R^2^ was 0.999, and the limit of detection was 0.014 µg mL^–1^. No recovery experiments were performed with 3-keto-DON since the focus of this study was the degradation of DON and we only aimed to observe selected products as confirmation of microbial transformation.

A suspicious peak with the same mass signal but a slightly shorter retention time compared to DON was observed after 3 min. This peak was putatively assigned to 3-epi-DON based on the shorter retention time which has been reported in other studies (Vanhoutte et al. 2017; Zhai et al. 2019) and based on the mass signals fitting with the signals of DON. Concentrations were calculated by using DON as an external standard due to its structural similarity. Chromatograms were also scanned for expected target masses of de-epoxy-DON (279.1232 [M]^–^ negative mode and 281.1389 [M]^+^ positive mode) as it is another known DON transformation product but without any result.

### Dissipation of DON in function of time, spiking level, and soil type

**Fig. 1:**
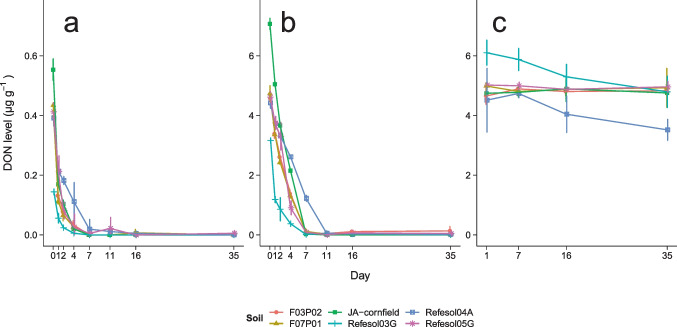
Mean levels and standard deviation (*n* = 3) of DON in various soils over time, at spiking levels 0.5 µg g^–1^ (**a**) and 5 µg g^–1^ (**b**) and in sterilized soils (**c**, 5 µg g^–1^)

**Fig. 2:**
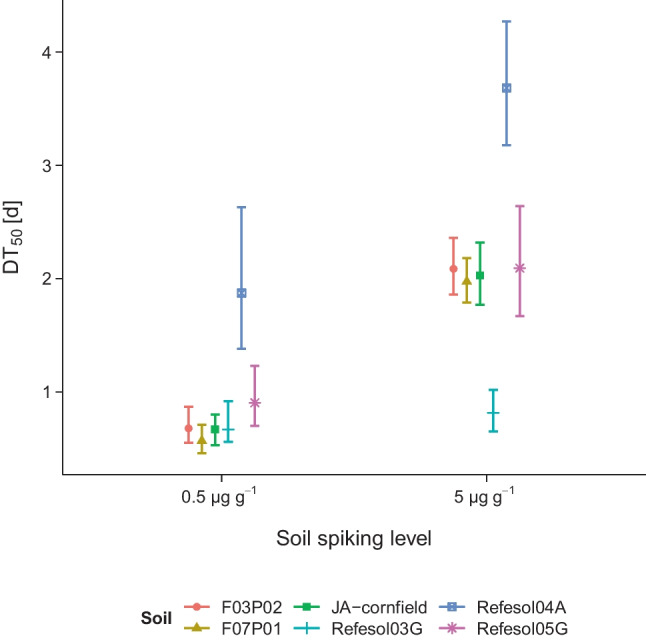
Comparison of DON dissipation in various soils at two spiking levels (0.5 and 5 µg g^–1^ soil dry weight). Values are estimated from simple first-order models and defined as the time when the initial level is reduced by 50%

In sterile soils, DON levels were constant over 35 days in most of the sterilized soils, but decreased by 21 ± 3% and 22 ± 6% in Refesol03G and Refesol04A within 35 days, respectively (Fig. 1c). Contrary to that, a fast dissipation of DON was observed in all non-sterilized soils and spiking levels (Fig. 1a and b). A considerably high initial DON level in JA-cornfield high spiking level was attributed to a spiking error where the volume of the spiking solution (50 $\upmu$L) was mixed up with the volume to adjust the WHC (75 $\upmu$L). Accordingly, the start level of the high spiking level of JA-cornfield was set to 7.5 µg g^–1^ prior to further evaluation. Within 4 days, almost all soil mean DON levels fell below 50% of the initially applied level, which is reflected in DT_50_ values between 0.57–1.87 days and 0.82–3.68 days for the low and high spiking level, respectively. Dissipation times estimated with simple first-order kinetic models (Table B1) were generally higher in the high spiking level compared to the low spiking level (Fig. 2).

DON levels appeared to increase in some soils of the high and low spiking levels after 10 days. For example, the mean DON level in F03P02 at day 7 was 0.07 ± 0.02 µg g^–1^ but 0.1 ± 0.2 µg g^–1^ at day 35 (see Fig. 1b). These seemingly higher levels are attributed to individual samples, which is also seen in the higher variation. However, no such increase was found in control samples that were not spiked with DON, and the levels in the low spiking level were usually below the limit of quantification after the seventh day. We therefore exclude within soil production of DON and interpret the values as variability between samples, potentially caused by the variability of the soil microbial community.

The slowest dissipation was observed in Refesol04A, irrespective of spiking level, while the fastest dissipation was observed in Refesol03G. The level in the first sampling of Refesol03G low spiking level was even below half of the initially spiked level.

### Formation of microbial transformation products in soil: 3-keto-DON and 3-epi-DON

**Fig. 3:**
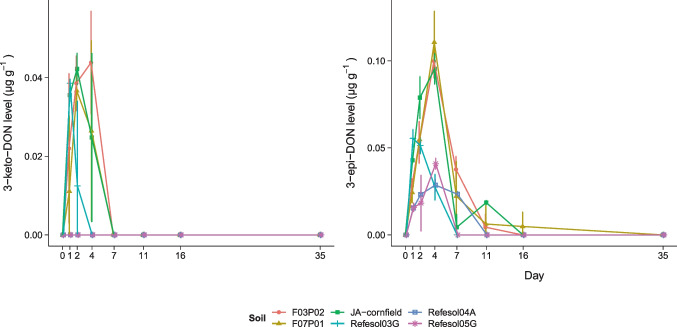
Mean levels with standard deviation (*n* = 3) of DON transformation products 3-keto-DON and 3-epi-DON in various soils after spiking with DON to 5 µg g^–1^

Degradation products of DON above respective limit of quantification were only found in the high spiking levels (Fig. 3). Levels of both transformation products rapidly increased within 4 days to 40 ng g^–1^ 3-keto-DON and 100 ng g^–1^ 3-epi-DON. Peak levels of 3-keto-DON were reached within 2 days and fell below the limit of quantification until the 7th day after DON application. Levels of 3-keto-DON in Refesol04A and Refesol05G were always below the limit of quantification.

Compared to 3-keto-DON, build-up of 3-epi-DON peak levels appeared slightly time-displaced, and quantifiable levels of 3-epi-DON were found up to 16 days after DON application. The initial build-up of DON transformation products in Refesol03G was fast followed by a fast decrease. The soils F03P02, F07P01, and JA-cornfield reached higher levels that were also detectable for a longer time. The levels of 3-epi-DON in Refesol04A and Refesol05G increased slower, and the levels were generally lower compared to the other soils.

### Relation of soil microbiology and DON residual levels

Soil respiration rate, measured as the release of CO_2_-C, varied between soils from 1.5 ± 0.2 ng h^–1^ g^–1^ soil in Refesol04A to 12 ± 2 ng h^–1^ g^–1^ soil in Refesol03G (Table 3). Similarly, the soil microbial biomass (total PLFA) was highly variable with the lowest content in F03P02 (4 ± 1 nmol g^–1^) and the highest in Refesol03G (22 ± 2 nmol g^–1^). Fractions of bacteria and fungi were less variable with 60–91% and 3–10%, respectively.

**Table 3:** Soil microbiological characteristics: glucose-induced respiration rate as nanogram CO_2_-C per gram soil and hour, measured 6 h after glucose addition. Total PLFA content as nano mol per gram soil, fungi, and bacteria as a molar percentage of total PLFA content. Fungi-to-bacteria ratio is the fraction of respective biomarker contents

Soil	Respiration rate	Total PLFA	Bacteria	Fungi	Fungi to bacteria
	ng CO_2_-C g^-1^ h^-1^	nmol g^-1^	mol%	mol%	F:B
F03P02	$2.6\pm 0.6$	$4\pm 1$	$80\pm 10$	$5\pm 2$	$0.09\pm 0.04$
F07P01	$4.0\pm 0.6$	$5\pm 1$	$80\pm 10$	$3\pm 2$	$0.06\pm 0.04$
JA-cornfield	$10\pm 1$	$8.6\pm 0.3$	$91\pm 1$	$9\pm 1$	$0.10\pm 0.01$
Refesol03G	$12\pm 2$	$22\pm 2$	$77\pm 5$	$6.5\pm 0.3$	$0.086\pm 0.009$
Refesol04A	$1.5\pm 0.2$	$9\pm 2$	$68\pm 9$	$8\pm 1$	$0.12\pm 0.02$
Refesol05G	$2.9\pm 0.4$	$13\pm 3$	$60\pm 8$	$10\pm 2$	$0.17\pm 0.02$

The microbial parameters were related to the residual DON levels at day 4, where also the highest mean level of transformation products was measured, indicative for microbial transformation activity. The relative residual level in the high spiking level after 4 days was significantly greater compared to the low spiking level (one-sided Wilcoxon rank sum test: *W* = 294, *p* < 0.01).

**Fig. 4:**
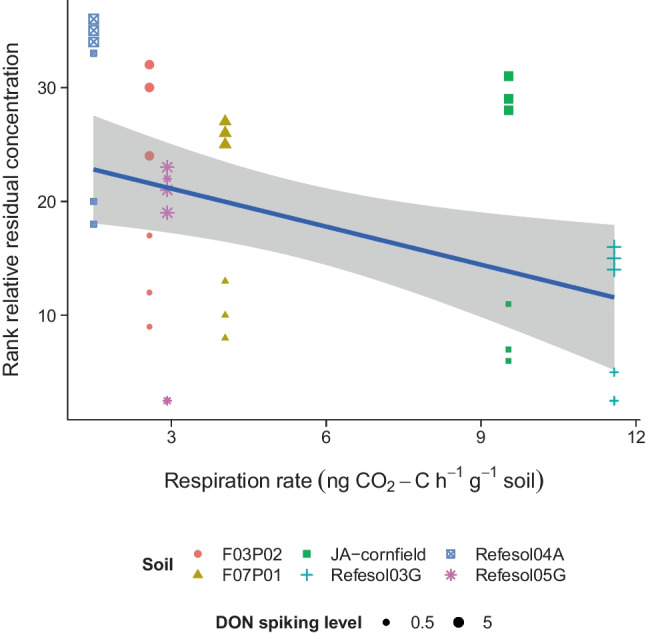
Correlation of ranked relative residual levels of DON and respiration rate in various soils, spiked with different levels of DON (0.5 and 5 µg g^–1^). Depicted in blue is a trendline with 95% confidence interval (slope *p* value < 0.01, R^2^ = 0.17)

The linear mixed effect model showed a significant effect of microbial respiration on residual level (numerator/denominator DF = 1/33, *F* value = 16.316, *p* value < 0.01), visible in a trend towards lower residual level with increasing microbial respiration (Fig. 4). The second linear mixed effect model including “fungi-to-bacteria ratio” and “soil microbial biomass” indicated significant effects of both factors with a trend towards ranked residual DON levels decreasing with “soil microbial biomass” and increasing with “fungi-to-bacteria ratio” (Table 4). Additionally, there was a significant interaction of both factors.

**Table 4:** Type II analysis of variance table according to Kenward-Roger’s method for factors soil microbial biomass * fungi-to-bacteria (FB) ratio on residual DON level in soil after 4 days

	Sum Sq	Mean Sq	Num DF	Den DF	*F* value	*p* value
Soil microbial biomass	141.96	141.96	1	31	3.5793	0.045
F:B ratio	362.40	362.40	1	31	10.7457	0.002
Soil microbial biomass:FB ratio	292.28	292.28	1	31	8.2593	0.005

## Discussion

### Dissipation of DON in agricultural soils

Compared to sterile soil samples, DON levels in non-sterilized samples clearly decreased with time. Within 24 h, an overall mean of 50 ± 20% of the initially applied DON level disappeared and only trace levels persisted for more than 10 days. We therefore attribute the decreasing DON levels to microbially determined processes, i.e., microbial transformation.

Although only 2.5 g soil dry weight were used in each sample, dissipation of DON was observed in all non-sterilized samples indicating that DON-transforming microorganisms were well distributed within the homogenized soil. However, we observed some variability in DON levels within individual sampling times particularly in the low spiking level. Besides the variability caused by the extraction and measurement method, this variability may also have biological reasons. Soil aggregates containing hot spots of microbial activity or diversity (Upton et al. 2019) may have a great influence on the DON transformation in the individual samples. However, the observed outliers were accepted as the variability of biological systems and included in the data evaluation where they did not obviously affect the observed trends.

The transformation in non-sterilized soils observed here was faster compared to studies, where DON was incorporated with plant material (Abid et al. 2021; Meyer-Wolfarth et al. 2021), most likely because DON dissolved in soil solution has a higher availability to soil microorganisms. Therefore, DON appears to be quickly degraded only when it is washed off from plants or released from plant material. When DON was applied as a part of plant material (i.e., straw or maize stubbles), DON was still detected several weeks after application (Abid et al. 2021; Meyer-Wolfarth et al. 2021). Soil fauna may play an important role in this process as indicated by Abid et al. (2021) where DON degradation was faster when DON-containing straw was mixed with soil and when earthworms were present. Similarly, Meyer-Wolfarth et al. (2021) showed faster DON dissipation in the presence of springtails and earthworms. They suggest that the microbial community may also indirectly contribute to the dissipation of DON in plant material by increasing the palatability of plant material for earthworms and springtails. The dissipation of DON in the low spiking level was clearly faster compared to the high spiking level as seen in the relative residual DON levels and the estimated DT_50_ values. This suggests that the transformation of DON in soil is level-dependent which contradicts the simple first-order kinetic. Therefore, simple first-order kinetic may not be suitable for DON dissipation modeling in soils, and DT_50_ values have to be interpreted carefully. Bekins et al. (1998) showed that degradation of organic pollutants at high levels may be better modeled by zero-order kinetics, and in fact, the dissipation of the high DON spiking level appeared to be linear in some soils (Fig. 1, e.g., JA-cornfield, Refesol04A). However, the intention behind the high spiking level was not to estimate dissipation times but to confirm microbial degradation via the occurrence of specific transformation products. The spiking level was set about three orders of magnitude above levels observed in natural soil samples (e.g., 2–28 ng g^–1^ reported in Muñoz et al. (2015, 2017)), and therefore, the low spiking level dissipation times may have greater practical relevance. In order to keep comparability of DT_50_ values, we only applied simple first-order models for value estimation.

Most of the soils in this study had similar DT_50_ values (Fig. 2) and residual levels within the respective spiking concentrations, which also indicates a similar DON transformation potential of soils independent of the physicochemical properties. However, two soils deviated particularly within the first 10 days: DON transformation in Refesol03G appeared to be faster compared to all other soils, while transformation in Refesol04A appeared to be slower. The effects of temperature, pH, and oxic state on the DON transformation potential of biological systems have already been shown (Ahad et al. 2017; Islam et al. 2012; Meyer-Wolfarth et al. 2021) but do not apply in this study since parameters were similar for both soils. However, the difference between Refesol03G and Refesol04A in DON transformation is reflected in the formation of transformation products (Fig. 3) and, interestingly, most of the soil microbial parameters (Table 3).

In the sterilized control, four out of six soils showed a constant DON level throughout the experiment. The transformation of DON via exoenzymes has already been demonstrated earlier by Shima et al. (1997). Considering the high stability of DON under various physical and chemical conditions (Feizollahi and Roopesh 2022), it seems most likely that exoenzymes persisted in the sterilization process and caused a slight disappearance of DON as observed in soils in this study. However, neither 3-epi-DON nor 3-keto-DON was observed in sterile samples, potentially because the transformation product levels were below the detection limit. Alternatively, factions of DON may be bound to soil organic matter, but this would be contradictory to previous studies confirming the low affinity of DON to soil organic matter (Schenzel et al. 2012b). Considering the fast dissipation of DON in non-sterilized soils, abiotic processes appear to be of less relevance compared to microbial transformation processes.

### The role of soil microbiology in degradation processes

Our study confirms, that one microbial transformation path of DON in soil is via the formation of 3-keto-DON and 3-epi-DON. This has been observed in DON detoxification experiments with soil microbial communities and strains in artificial medium (Yao and Long 2020), but never in situ. Ahad et al. (2017) and Islam et al. (2012) applied DON to a soil microbial culture in soil extract medium, but observed no de-epoxidation activity, while the same cultures in mineral salt media were able to de-epoxidize DON. Expected target masses of de-epoxidized DON were not found in this study although aerobic de-epoxidation by microorganisms from crop fields was confirmed in incubation studies (Islam et al. 2012). Therefore, under the conditions of this study, de-epoxidation may be interpreted as a non-relevant detoxification pathway compared to the bacterial epimerization of DON. Hassan et al. (2017) showed that the enzymatic epimerization of DON proceeds through the formation of 3-keto-DON as an intermediate. Similarly, we observed time-displaced maximum levels of 3-keto-DON prior to 3-epi-DON.

Hassan et al. (2017) also reported an accumulation of transformation products, which was not observed here. The sum of transformation products was never exceeding 3.5% of the initially applied DON level and both products were not detectable after 16 days. This suggests that further transformation paths occur in soil leading to unknown products, mineralization, or microbial incorporation as a carbon source (Zhai et al. 2019; Venkatesh and Keller 2019; Sato et al. 2012).

Interestingly, the soils sampled from corn fields (F03P02, F07P01, JA-cornfield) showed similar transformation product levels over time. Additionally, the slowest transformation and lowest levels of transformation products were observed in the soil sampled from arable land (Refesol04A). The sample size of six soils taken from three different land managements does not allow conclusions about priming effects of land management on DON transformation potential of soils. However, our results indicate that there may be differences based on land management and provide useful information about the optimal sampling time interval (1–4 days) after application, to assess such effects in further studies using larger sample sizes.

Dissipation of DON was observed in all investigated soils. Even in soils which were frozen between field sampling and pre-incubation, i.e., F03P02 and F07P01, DON levels dropped by over 90% within less than 10 days. Although the soils in the sampling area are regularly frozen during winter and freezing at $-$20 ${}^{\circ }\text {C}$ may not have changed the microbial biomass and respiration (Stenberg et al. 1998), some microbial strains may have been affected by the storage. However, these soils were included in this study because of the previous crop culture (maize) and presence of DON as proven in a previous study (Kenngott et al. 2022). At least in these soils, the potential of soil microbial communities to transform DON seems not to be affected by the storage of samples indicating that DON transformation is a more resilient property of the community. This is contrary to studies where only a few soil microbial communities extracted from various soils were able to degrade DON in artificial media (Völkl et al. 2004; Islam et al. 2012). These contrasting findings may be explained by the fact that many soil microbial species are specialized to soils and can not be cultivated under laboratory conditions or in artificial media (Ward et al. 1990; Völkl et al. 2004; Hengstmann et al. 1999). Therefore, the cultivable microbial community of soil extracts may have lost the microbial strains bearing the DON transformation potential. Our results indicate, that the ability to degrade DON is much more common in soil microbial communities than could be expected from soil extract studies.

There was a significant trend towards lower residual levels, i.e., faster DON transformation, in soils with high microbial respiration indicative of a high microbial activity. This was particularly pronounced in Refesol03G and Refesol04A, where an eight-fold difference in respiration and a four-fold difference in DT_50_ were observed. Additionally, there was a trend towards faster transformation of DON in soils with greater microbial biomass and lower fungi-to-bacteria ratio. However, the interaction of both factors indicates that this has to be interpreted carefully and that the transformation of DON may be accelerated by greater microbial biomass, when the fraction of fungi is increased. The observation of a positive relation between soil microbial biomass and transformation is in line with Anderson (1984), where the same effect was observed for herbicide degradation in a soil with altered microbial biomasses. Our results indicate that the ability to degrade DON is distributed among several species within the soil microbial community and a greater microbial biomass would consequently have a greater reactive surface and degrade a larger fraction of dissolved DON. Additionally, transformation of DON was more often observed for bacterial strains than fungi (Yao and Long 2020). Although this may be biased by the experimental designs used in these studies, it indicates that bacteria may have a greater contribution to the transformation potential of soil microbial communities. This corresponds to the finding of this study which shows a trend towards faster transformation with decreasing fungi-to-bacteria ratio.

Soils may generally contribute to DON detoxification via microbial transformation to 3-keto-DON and 3-epi-DON. Further unknown transformation products and additional biogeochemical pathways, i.e., mineralization should be investigated in further studies. The transformation rate observed in this study was fast, i.e., hours to days. This may also explain the low number of positive maize field soil samples (5 out of 64) found in Kenngott et al. (2022), where samples were taken at the time of harvest, but not after rain events when DON may be detected. However, the fast dissipation observed here raises the question of how elevated environmental concentrations in soil leachate and surface waters can be observed (Schenzel et al. 2012a, c; Kolpin et al. 2014). One explanation could be that DON transport through soil was too fast during intense rain events or that the main route of rainwater was via surface runoff rather than soil infiltration. It is also possible that other factors, which have not been included in this study, e.g., different temperature (Meyer-Wolfarth et al. 2021) or soil moisture, may either enhance or restrict the transformation activity. As shown in this study, very high levels of DON in soil are degraded slower, and fields with high *Fusarium* infestation and subsequent high DON levels may cause this emission of DON to surface waters. The occurrence of DON in the environment and the ecological effects may be investigated in future studies, and our results may help to adapt sampling strategies to detect DON levels in soils. The influence of land management on the transformation potential of soils remains unclear, but further studies with larger sample size may detect priming effects as indicated in this study. Although there is a trend towards faster DON transformation in soils with large and active soil microbial communities and a greater bacterial fraction, the transformation remained variable among the investigated soils and general soil microbial community characteristics appear not to be sufficient for explaining the transformation potential. Our results indicate that the ability to degrade DON is much more abundant in soil microbial communities than it can be expected based on previous studies. Our study provides the first insight to in situ microbial DON transformation in agricultural soils. This is relevant in the context of mycotoxins as environmental pollutants of emerging concern and shows the role of soils to mitigate environmental DON levels.

## Electronic supplementary material

Below is the link to the electronic supplementary material.

Supplementary file1 (DOCX 932 KB)

## Data Availability

The datasets generated during and analyzed during the current study as well as the code are available from the corresponding author on reasonable request.
