# Supplementary material for: The potential of soil microbial communities to transform deoxynivalenol in agricultural soils—a soil microcosm study
Source: Mycotoxin Res. 2024 Mar 20;40(2):295–307. doi: 10.1007/s12550-024-00526-5 (PMC11588787; doi:10.1007/s12550-024-00526-5)
Supplement: Supplementary file 1 — Supplementary file1 (DOCX 932 KB) [file 12550_2024_526_MOESM1_ESM.docx]

**Appendix A Additional figures**


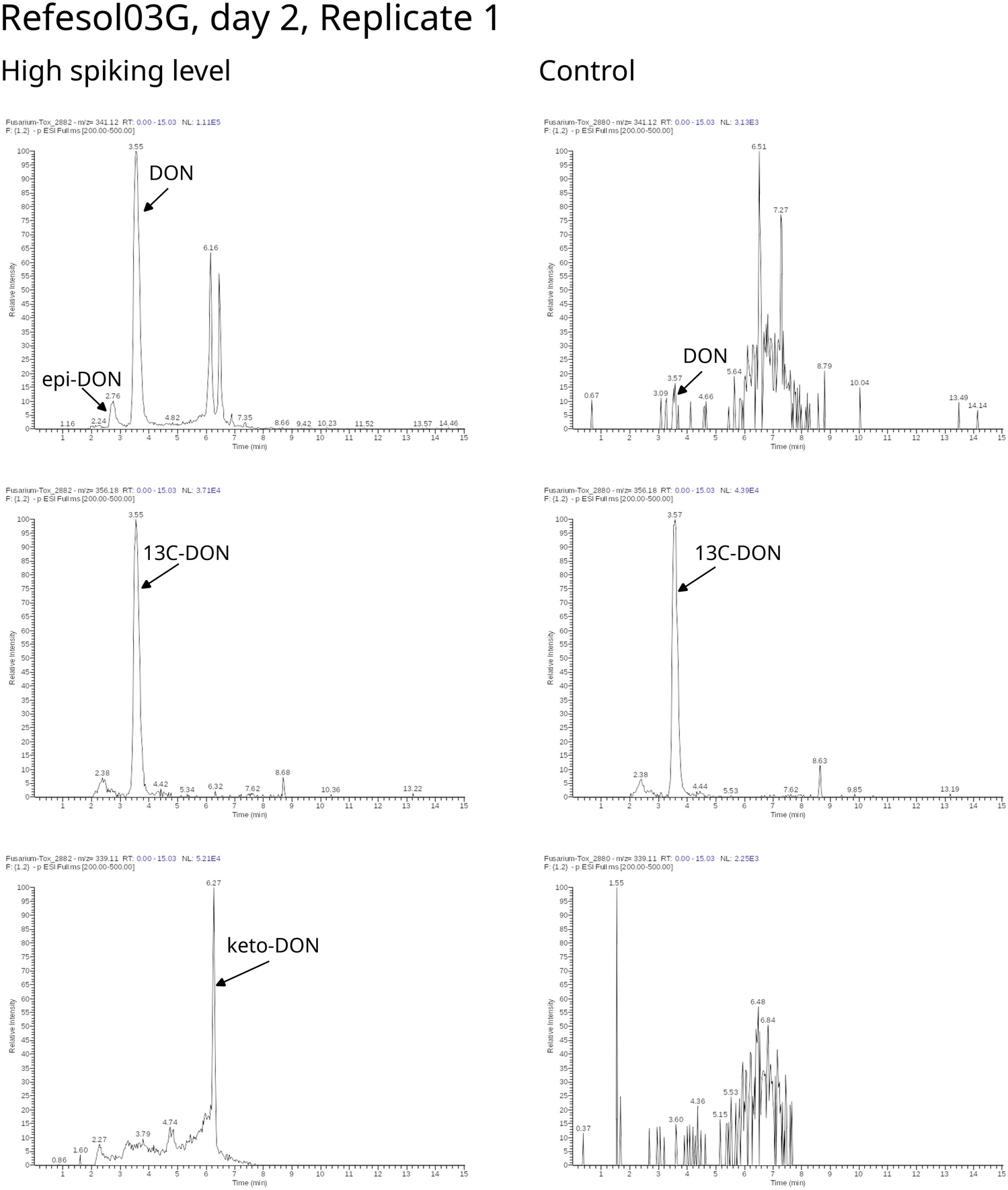


Fig. A1 Chromatogram examples of 3-epi-DON, DON, isotopically labeled DON (13C-DON, internal standard) and keto-DON, measured two days after DON application in the first replicate of Refesol03G high spiking level (5 µg g^−1^, left column) and the respective control (0 µg g^−1^, left column). Traces of DON in the control were below the limit of quantification and identified as carryover.


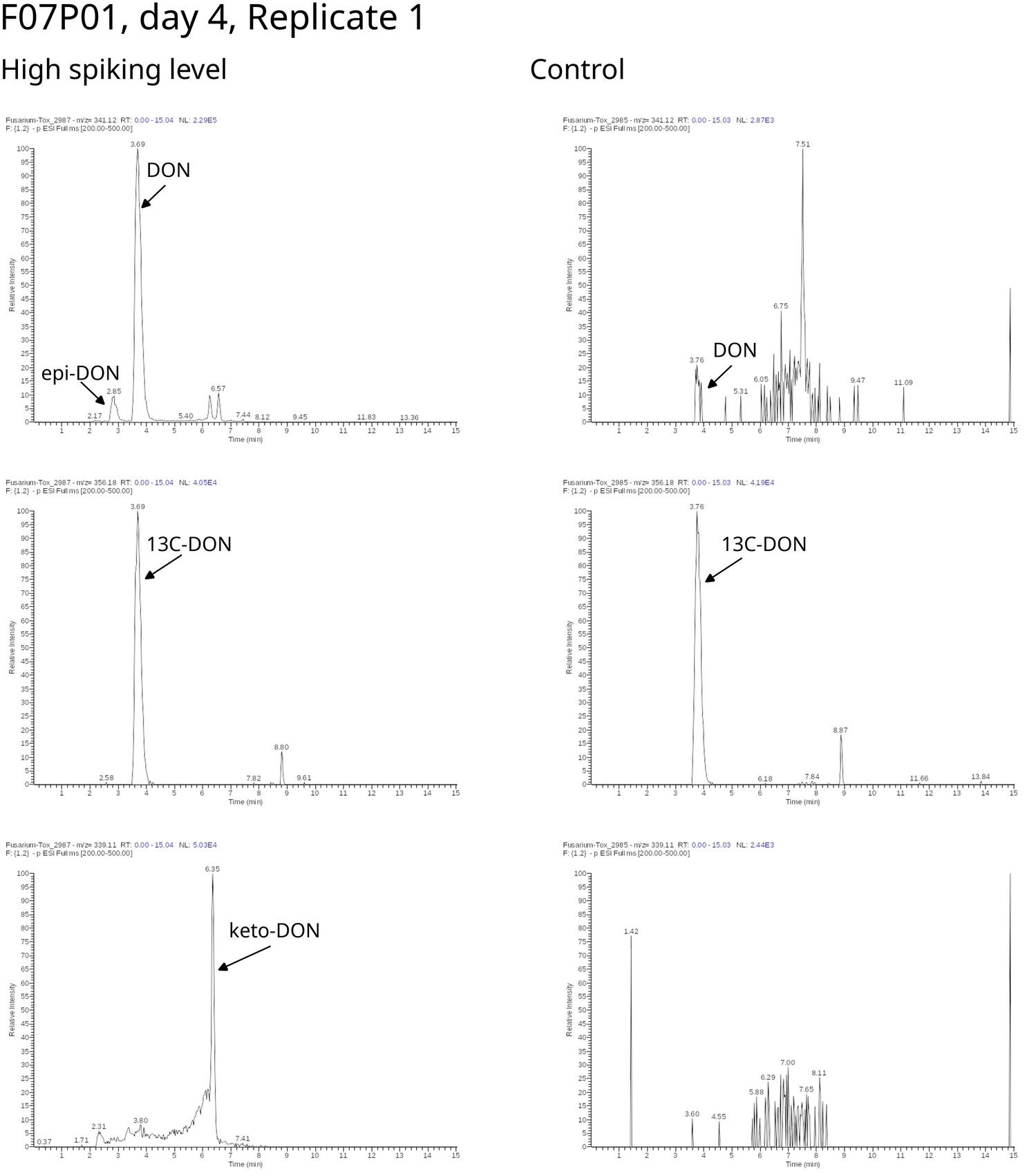


Fig. A2 Chromatogram examples of 3-epi-DON, DON, isotopically labeled DON (13C-DON, internal standard) and keto-DON, measured two days after DON application in the first replicate of F07P01 high spiking level (5 µg g^−1^, left column) and the respective control (0 µg g^−1^, left column). Traces of DON in the control were below the limit of quantification and identified as carryover.

**Appendix B Additional tables**

**Table B1** Estimated simple first order parameters of DON in various soils at two spiking levels: initial concentration (C_0_), dissipation rate konstant (k) and estimated 50% dissipation times (DT50) with 95% confidence interval (CI).

| Spike level | Soil | C_0_ | k | DT50 | Lower CI | Upper CI |
| --- | --- | --- | --- | --- | --- | --- |
| µgg−1 |  | µgg−1 | d−1 | d | d | d |
| 5 | F03P02 | 4.76 | 0.33 | 2.09 | 1.86 | 2.36 |
| 5 | F07P01 | 4.80 | 0.35 | 1.97 | 1.79 | 2.18 |
| 5 | JA-cornfield | 7.13 | 0.34 | 2.03 | 1.77 | 2.32 |
| 5 | Refesol03G | 3.48 | 0.85 | 0.82 | 0.65 | 1.02 |
| 5 | Refesol04A | 4.65 | 0.19 | 3.68 | 3.18 | 4.27 |
| 5 | Refesol05G | 5.07 | 0.33 | 2.09 | 1.67 | 2.64 |
| 0.5 | F03P02 | 0.43 | 1.02 | 0.68 | 0.55 | 0.87 |
| 0.5 | F07P01 | 0.44 | 1.23 | 0.56 | 0.46 | 0.70 |
| 0.5 | JA-cornfield | 0.55 | 1.03 | 0.67 | 0.53 | 0.80 |
| 0.5 | Refesol03G | 0.17 | 1.04 | 0.67 | 0.56 | 0.92 |
| 0.5 | Refesol04A | 0.38 | 0.37 | 1.87 | 1.38 | 2.63 |
| 0.5 | Refesol05G | 0.45 | 0.77 | 0.90 | 0.70 | 1.23 |
